# Development of an integrated predictive model for postoperative glioma-related epilepsy using gene-signature and clinical data

**DOI:** 10.1186/s12885-022-10385-x

**Published:** 2023-01-11

**Authors:** Lianwang Li, Chuanbao Zhang, Zheng Wang, Yinyan Wang, Yuhao Guo, Chong Qi, Gan You, Zhong Zhang, Xing Fan, Tao Jiang

**Affiliations:** 1 Department of Neuro-Oncology and Neurosurgery, Tianjin Medical University Cancer Institute and Hospital, Tianjin, 300060 China; 2 Department of Neurosurgery, Beijing Tiantan Hospital, Capital Medical University, Beijing, 100070 China; 3 Beijing Neurosurgical Institute, Capital Medical University, Beijing, 100070 China; 4 Research Units of Accurate Diagnosis and Treatment of Brain Tumors and Translational Medicine, Chinese Academy of Medical Sciences, Beijing, 100730 China

**Keywords:** Glioma-related epilepsy, Diffuse high-grade gliomas, Gene-signature, Clinical data, Integrated prediction model

## Abstract

**Background:**

This study aimed to develop an integrated model for predicting the occurrence of postoperative seizures in patients with diffuse high-grade gliomas (DHGGs) using clinical and RNA-seq data.

**Methods:**

Patients with DHGGs, who received prophylactic anti-epileptic drugs (AEDs) for three months following surgery, were enrolled into the study. The patients were assigned randomly into training (*n* = 166) and validation (*n* = 42) cohorts. Differentially expressed genes (DEGs) were identified based on preoperative glioma-related epilepsy (GRE) history. Least absolute shrinkage and selection operator (LASSO) logistic regression analysis was used to construct a predictive gene-signature for the occurrence of postoperative seizures. The final integrated prediction model was generated using the gene-signature and clinical data. Receiver operating characteristic analysis and calibration curve method were used to evaluate the accuracy of the gene-signature and prediction model using the training and validation cohorts.

**Results:**

A seven-gene signature for predicting the occurrence of postoperative seizures was developed using LASSO logistic regression analysis of 623 DEGs. The gene-signature showed satisfactory predictive capacity in the training cohort [area under the curve (AUC) = 0.842] and validation cohort (AUC = 0.751). The final integrated prediction model included age, temporal lobe involvement, preoperative GRE history, and gene-signature-derived risk score. The AUCs of the integrated prediction model were 0.878 and 0.845 for the training and validation cohorts, respectively.

**Conclusion:**

We developed an integrated prediction model for the occurrence of postoperative seizures in patients with DHGG using clinical and RNA-Seq data. The findings of this study may contribute to the development of personalized management strategies for patients with DHGGs and improve our understanding of the mechanisms underlying GRE in these patients.

**Supplementary Information:**

The online version contains supplementary material available at 10.1186/s12885-022-10385-x.

## Background

Epilepsy is a common neurological disorder characterized by unprovoked seizures that affects approximately 70 million people worldwide [1]. Structural abnormality is one of the main etiologies of epilepsy, among which brain tumor constitutes a critical cause [2]. As the most common primary intracranial malignant tumor, glioma has long been a research hotspot in the field of neurosurgery [3]. Glioma is also one of the most highly epileptogenic brain tumors, and glioma-related epilepsy (GRE) has become a focus for neurosurgeons [4]. GRE is the initial symptom in most patients with diffuse gliomas, and 15%–60% of the patients suffer from GRE even after craniotomy [2, 5]. To date, seizure control remains a challenge in the management of glioma.

The main reason for the increased concern over GRE is its adverse impact on the quality of life of patients following tumor resection. Studies have focused on GRE associated with diffuse low-grade gliomas (WHO grade 2) due to the higher incidence of GRE and the longer survival of patients with this form of glioma [6, 7] while studies of GRE in diffuse high-grade gliomas (DHGGs, WHO grades 3 and 4) are rare. The confluence of tumor and epilepsy can disrupt the daily life of patients and further reduce their quality of life [8]. In order to improve postoperative seizure control in DHGG patients, early selective application of anti-epileptic drugs (AEDs) is warranted, which necessities the identification of the patient group with a high-risk of postoperative seizure occurrence. Predictive postoperative seizure outcome studies in patients with DHGG are of great significance in this regard.

We previously conducted a comprehensive epidemiological study of GRE in patients with DHGGs and established a clinical feature-based prediction model (predictive nomogram) for seizure outcomes [9]. A clinical feature-based model has the advantage of high generalizability and convenience, although its accuracy is often limited. Advances in high-throughput RNA-sequencing technologies have enabled the development of RNA-seq-based clinical prediction models with higher accuracies. RNA-seq-based prediction models of seizure outcomes in patients with diffuse low-grade gliomas have been generated [10, 11], but similar models have not been generated for patients with DHGGs. This study aimed to use RNA-Seq and clinical data to develop a prediction model for postoperative seizure occurrence in DHGG patients and to identify possible mechanisms underlying GRE.

## Materials and methods

### Patient characteristics and data collection

Data from 208 patients who underwent craniotomy and were diagnosed with primary DHGG at Beijing Tiantan Hospital, China, were retrospectively reviewed. Patients who previously underwent surgery for other intracranial diseases or had any other concomitant malignant diseases were excluded from the study. All patients had received the standard treatment, including maximal safe resection and adjuvant radiotherapy and/or chemotherapy, depending on their tumor grade. Demographic data (age and gender) and the main complaints from patients were retrieved from an electronic medical record system. Tumor samples collected during craniotomy were flash frozen in liquid nitrogen and stored at -80 °C. Hematoxylin and eosin staining was performed, and tumor tissues containing > 80% tumor cells were selected for further analysis. Molecular pathological characteristics, including IDH1/2 mutation, chromosome 1p19q co-deletion, and MGMT promoter methylation status were assessed through pyrosequencing or fluorescence, as previously described [12, 13]. The pathological diagnosis of each patient was re-evaluated by integrating histopathology and molecular biomarkers according to the 2016 WHO classification of tumors of the central nervous system[14]. Transcriptomic data of samples collected during surgery were generated on the Agilent Whole Human Genome Array platform, as previously described [15]. RNA expression data were converted into fragments per kilobase million matrix and used in subsequent analysis.

Preoperative GRE was defined as at least one unprovoked epileptic seizure prior to surgery based on the patients' main complaint and history of illness. All patients received prophylactic AEDs after surgery, including phenobarbital injections administered within 3 d and valproate or levetiracetam tablets administered for at least 3 months. Seizure outcome was followed up via telephone interviews conducted 12 months after surgery, and postoperative seizure occurrence was defined as at least one unprovoked epileptic seizure occurring between the day of discharge and the day of follow-up. Patients with overall survival time of less than 12 months were excluded from the study. All data analyzed here, except those related to seizures, have been uploaded to the CGGA portal (http://www.cgga.org.cn/) for open access [16].

### Identification of differentially expressed genes and enrichment analysis

Patients were randomly assigned to either a training or validation cohort in a 4:1 ratio. The prediction model was established using the training cohort and validated using the validation cohort. The R package “Limma” was used to screen for differentially expressed genes (DEGs) between patients with or without preoperative GRE under the criteria of adjusted *p*-value < 0.05 (Benjamini and Hochberg Method) and |log2(fold change)|≥ 0.8. Gene Ontology (GO) enrichment analysis, Kyoto Encyclopedia of Genes and Genomes (KEGG) enrichment analysis[17–19], and Gene Set Enrichment Analysis (GSEA) were conducted using the R package “clusterProfiler.” Adjusted *p* < 0.05 was used as the cut-off threshold. Enrichment analyses used the Molecular Signatures Database (MSigDB) as a reference.

### Assessment of tumor-infiltrating immune cells

Immune cell infiltration in tumor samples was analyzed using the “CIBERSORT” algorithm (https://cibersort.stanford.edu/), which was used to evaluate the cell composition of tumor samples based on gene expression profiles. The proportions of 22 types of tumor-infiltrating immune cells were evaluated according to the LM22 signature genes file, with 1000 permutations. Tumor infiltration levels in patients with and without preoperative GRE were compared.

### Identification of a predictive gene-signature of postoperative seizure occurrence

*The DEGs were used to construct a gene-signature for postoperative seizure* occurrence using logistic regression, regularized with the least absolute shrinkage and selection operator (LASSO) algorithm. The analysis was conducted using the R package “glmnet.” The risk score for each individual was calculated using the formula:

Risk score = $$\sum_{{\varvec{i}}=1}^{{\varvec{n}}}{{\varvec{\beta}}}_{{\varvec{i}}}{{\varvec{x}}}_{{\varvec{i}}}$$(1)

Where, x represents the expression level of genes selected to construct the gene-signature, and *β* represents the corresponding regression coefficients.

The performance of the risk score was evaluated using receiver operating characteristic (ROC) analysis and the area under the curve (AUC), implemented using the R package “pROC.” Patients in the training and validation cohorts were then divided into high-risk and low-risk groups based on the median risk score value.

### Weighted correlation network analysis

Weighted correlation network analysis (WGCNA), implemented using the R package “WGCNA,” was conducted to identify key modules and genes with the highest correlation with preoperative GRE history, postoperative seizure occurrence, and the obtained gene-signature. Age, WHO grade, IDH1/2 mutation status, chromosome 1p/19q codeletion status, preoperative GRE history, postoperative seizure occurrence, and risk scores were included as variables in the analysis. Risk scores and patient age (with a cut-off of 45 years) were included as dichotomous categorical variables. The top 5000 genes (based on variance) were screened, and those with *β* = 5 (R^2^ ≥ 0.9) were selected and used to construct an unsigned scale-free co-expression gene network. The smallest module contained at least 30 genes, and analogous modules were fused at a height cut-off of 0.25. Genes in the obtained key modules were extracted for further analysis.

### Identification of hub genes

WE generated a new set of DEGs based on risk scores (RDEGs) by identifying genes that were differentially expressed between high-risk and low-risk groups and selecting genes with potential effect on both the occurrence and outcome of GRE. Genes overlapping upregulated or downregulated RDEGs and genes extracted from WGCNA were selected. Protein–protein interaction (PPI) analysis was performed using the R package “STRINGdb” to explore the interactions among these genes. Genes showing high numbers of PPI interactions (those interacting with more than five other nodes) were identified as hub genes. The expression levels of hub genes in the validation cohort were also evaluated.

### Construction of a prediction model for postoperative seizure occurrence

A multivariate logistic regression analysis of clinical and gene-signature data was conducted to identify the independent risk factors associated with postoperative seizure occurrence. Statistically significant factors (*p* < 0.05) were included in the final prediction model, and a corresponding predictive nomogram for poor seizure control was constructed using the R package “rms.” Finally, ROC analysis and AUCs were used to evaluate the accuracy of the final prediction model using the training and validation cohorts.

### Statistical analysis

Statistical analysis was performed using R software version 4.0.5 (R Foundation for Statistical Computing, Vienna, Austria, www.r-project.org). Figures were generated using R packages, including “ggolot,” “pheatmap,” and “ggraph.” Chi-square and Fisher’s exact tests were used to evaluate the statistical significance of categorical variables. Patients with missing data were excluded from multivariate analyses. Statistical significance was set at *p* < 0.05, unless otherwise stated.

## Results

### Baseline patient characteristics

The 208 patients enrolled into the study were randomly assigned into a training cohort (*n* = 166) and a validation cohort (*n* = 42). Detailed information on the patients are presented in Table 1. Of the patients, 77 had a history of preoperative GRE (62 in the training cohort and 15 in the validation cohort), while postoperative seizures occurred in 37 patients (30 in the training cohort and 7 in the validation cohort) 12 months after surgery. We further compared the baseline characteristics between the enrolled patients and excluded patients, who died within 12 months, and found many variables including age at diagnosis, WHO grade, tumor pathology, incidences of pre- and postoperative GRE were significantly different.

**Table 1:** Baseline characters of patients enrolled in this study

Variable	Training cohort	Validation cohort
Total number	166	42
Gender		
Male	101 (60.8%)	19 (45.2%)
Female	65 (39.2%)	23 (54.8%)
Age at diagnosis (years)	45 (14–72)^a^	44 (19–68) ^a^
< 45	88 (53.0%)	18 (42.9%)
≥ 45	78 (47.0%)	24 (57.1%)
Tumor side		
Left	71 (42.8%)	21 (50.0%)
Right	87 (52.4%)	17 (40.5%)
Bilateral	7 (4.2%)	4 (9.5%)
Frontal lobe involvement		
Involved	105 (63.3%)	27 (64.3%)
None	61 (36.7%)	15 (35.7%)
Temporal lobe involvement		
Involved	73 (44.0%)	13 (31.0%)
None	93(56.0%)	29 (69.0%)
Neuro-function deficit		
Yes	46 (27.7%)	16 (38.1%)
No	120 (72.3%)	26 (61.9%)
Preoperative GRE		
GRE	62 (37.3%)	15 (35.7%)
Non-GRE	104 (62.7%)	27 (64.3%)
Postoperative GRE		
GRE	30 (18.1%)	7 (16.7%)
Non-GRE	136 (81.9%)	35 (83.3%)
EOR		
GTR	81 (48.8%)	26 (61.9%)
Non-GTR	85 (51.2%)	16 (38.1%)
WHO grade		
3	99 (59.6%)	27(64.3%)
4	67 (40.4%)	15 (35.7%)
Tumor pathology		
AA	68 (41.0%)	15 (35.7%)
AO	31 (18.7%)	12 (28.6%)
GBM	67 (40.3%)	15 (35.7%)
IDH1/2 mutation		
Mutation	83 (50.0%)	26(61.9%)
Wild type	83(50.0%)	16 (38.1%)
Chromosome 1p/19q codeletion^†^		
Codeletion	42 (25.3%)	13 (31.0%)
Non-codeletion	111 (66.9%)	28 (69.0%)
MGMT promoter^‡^		
methylated	81 (48.8%)	22 (52.4%)
unmethylated	61 (36.7%)	17 (40.5%)
Concurrent Radio-chemotherapy		
Yes	124 (74.7%)	29 (69.0%)
No	39 (23.5%)	13 (31.0%)

*GRE* glioma-related epilepsy, *EOR* extent of resection, *GTR* gross total resection, *AA* anaplastic astrocytoma, *AO* anaplastic oligodendroglioma, *GBM* glioblastoma, *IDH* isocitrate dehydrogenase, *MGMT* methylation of O6-methylguanine-DNA methyltransferase

^a^Average value (range)

^†^Data were available for 194 patients

^‡^Data were available for 181 patients

### Identification and enrichment analysis of DEGs

**Fig. 1:**
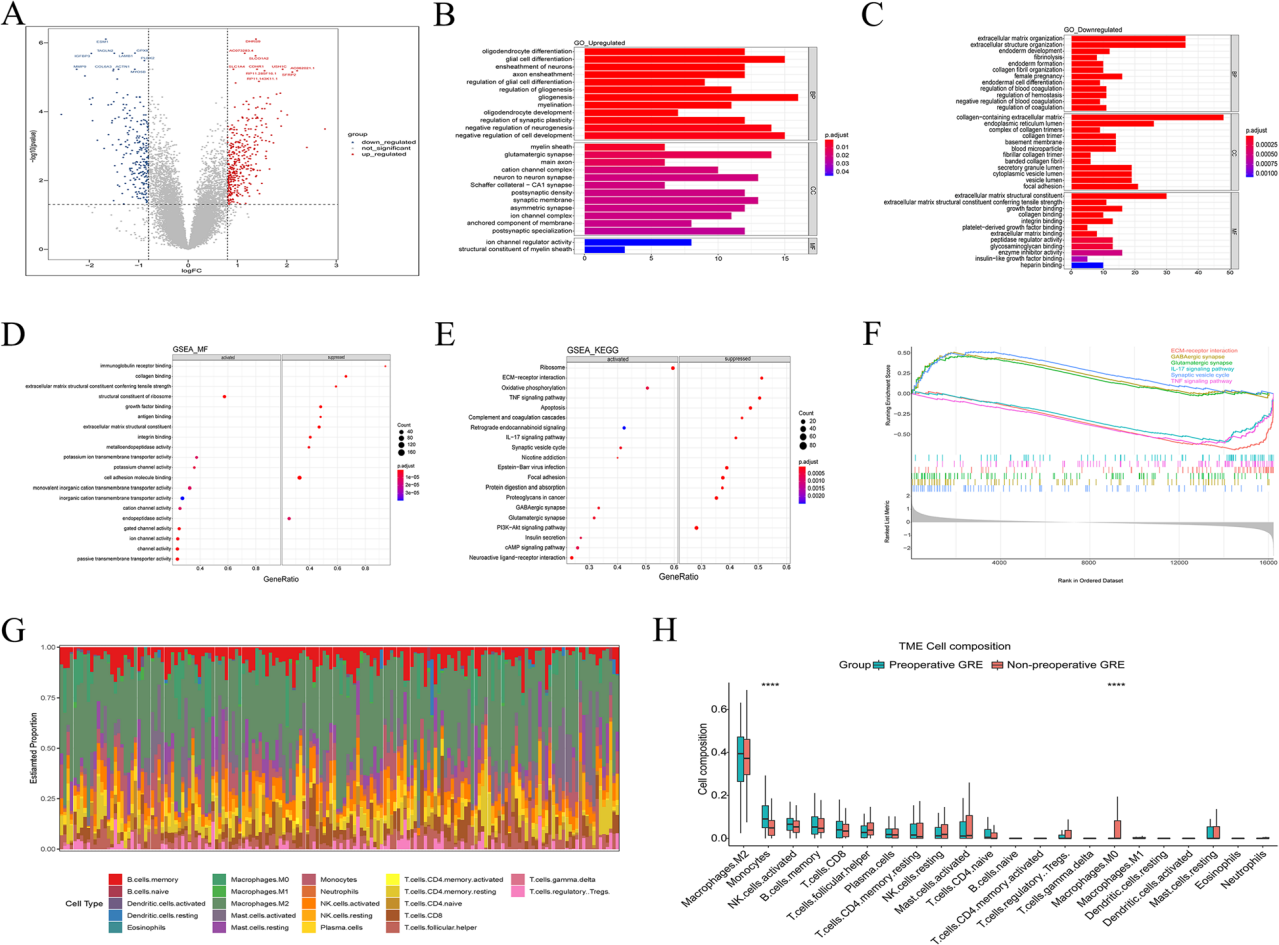
Identification and functional enrichment analysis of differentially expressed genes (DEGs) associated with preoperative glioma-related epilepsy (GRE). **A**, Volcano plot of DEGs identified based on adjusted *p* < 0.05 and |log2fc|≥ 0.8. (B-F), functional enrichment analysis of DEGs: **B**-**C**, Gene ontology (GO) terms associated with upregulated and downregulated genes; **D**-**F**, Enriched molecular functions and pathways associated with preoperative GRE history based on gene set enrichment analysis (GSEA). **G**-**H**, Distribution of 22 immune cells in the training cohort, and differences in tumor microenvironment (TME) cell compositions of patients with and without preoperative GRE

Based on the “Limma” algorithm, 623 DEGs were associated with preoperative GRE in the training cohort, including 381 upregulated and 242 downregulated genes (Fig. 1A). Functional enrichment analysis revealed that upregulated DEGs were mainly associated with ion channel regulator activity and structural constituent of myelin sheaths (Fig. 1B, Supplementary Fig. 1A, GO terms), and neuroactive ligand-receptor interaction and cAMP signaling pathway (Supplementary Fig. 1B, KEGG). Downregulated DEGs were predominantly involved in extracellular matrix organization and structure (Fig. 1C, Supplementary Fig. 1C, GO terms), and ECM-receptor interaction and focal adhesion (Supplementary Fig. 1D, KEGG). GSEA showed that ion channel activity (GO terms) and GABAergic synapse and glutamatergic synapse pathway (KEGG) were activated, while lymphocyte and adaptive immunity (GO terms) and ECM-receptor interaction and TNF signaling pathway (KEGG) were suppressed in patients with preoperative GRE (Fig. 1D-F). In addition, the relative proportions of 22 types of tumor-infiltrating immune cells were evaluated using the “CIBERSORT” algorithm. The landscape of immune cell infiltration is shown in Fig. 1G. The results showed that the levels of monocytes were significantly increased, while the levels of M0 macrophages were significantly lower in patients with preoperative GRE (Fig. 1H).

### Construction of a predictive gene-signature for postoperative seizure occurrence

**Fig. 2:**
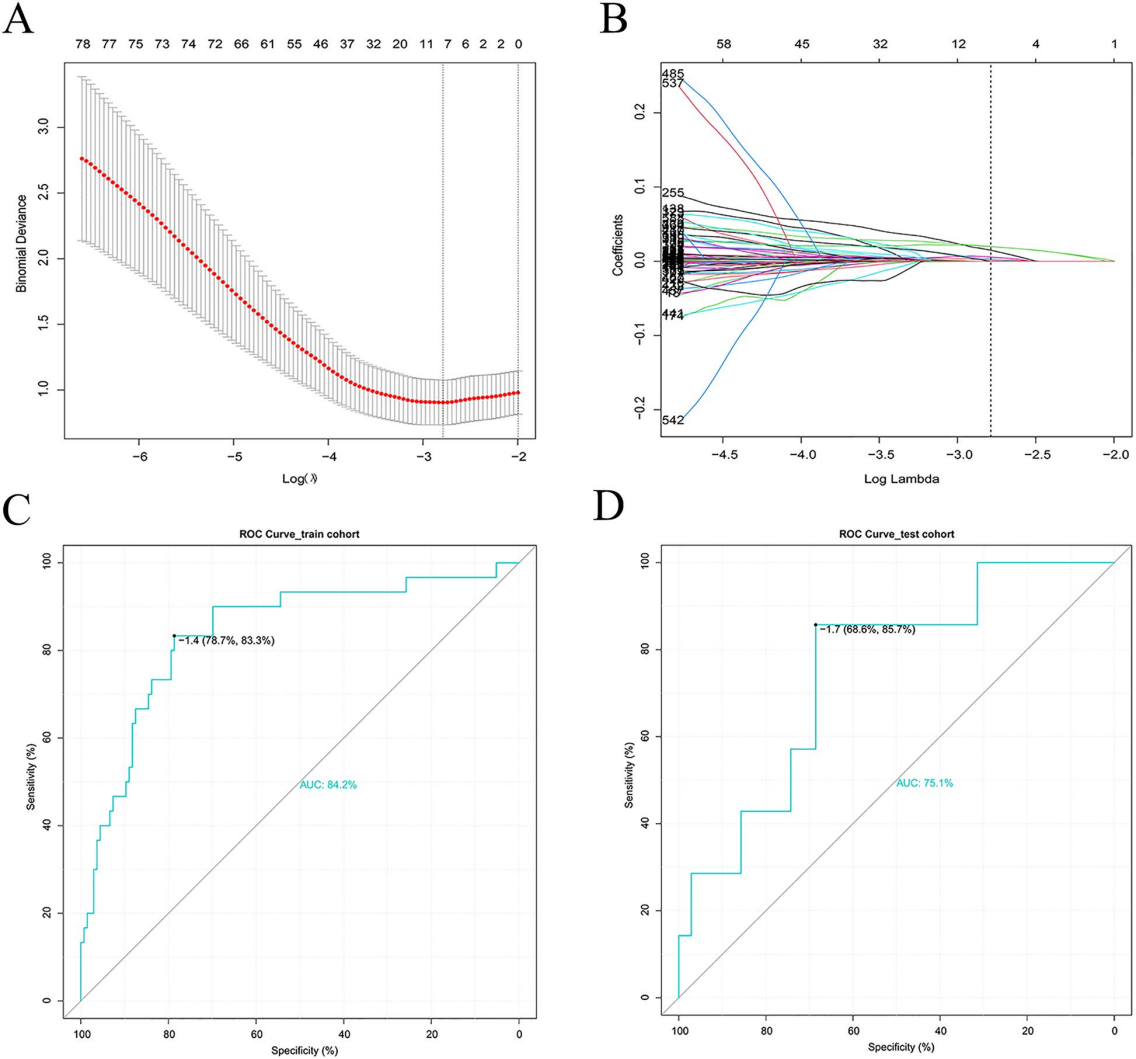
Construction and receiver operating characteristic (ROC) analysis of a gene-signature for postoperative seizure occurrence using Lasso logistic regression analysis. **A**-**B**, Lasso logistic regression analysis identified seven genes associated with postoperative seizure occurrence in the training cohort. The genes were used to construct the gene-signature. **C**-**D**, Determining the predictive accuracy of the gene-signature in the training and validation cohorts using ROC analysis

LASSO logistic analysis of DEGs was used to identify genes with the highest ability to predict postoperative seizure occurrence. Seven genes: *LUZP2, GPNMB, MMD2, GALNT13, IKBKGP1, SLC1A4*, and *RP1* were identified and subsequently used to construct a predictive gene signature (Fig. 2A-B). Detailed information on these seven genes is given in Table 2. The risk score for each individual was calculated using the following formula:

**Table 2:** Postoperative seizure occurrence associated genes identified via lasso regression

Gene Name	Gene Title	Gene Function	*β*
LUZP2	Leucine zipper protein 2	Extracellular matrix development	0.000647
GPNMB	Glycoprotein nmb	Cell–cell signaling	0.002348
MMD2	Monocyte to macrophage differentiation-associated 2	Protein kinase activity	0.003071
GALNT13	Polypeptide N-acetylgalactosaminyltransferase 13	Metal ion binding	0.005717
IKBKGP1	Inhibitor of nuclear factor kappa B kinase subunit gamma pseudogene 1	None	0.014818
SLC1A4	Solute carrier family 1 member 4	Synaptic transmission, glutamatergic	0.019691
RP1	RP1 axonemal microtubule associated	Intracellular signal transduction	0.019902

Risk score = 0.000646822 * Exp_LUZP2_ + 0.002348291 * Exp_GPNMB_ + 0.003071414 * Exp_MMD2_ + 0.005716866 * Exp_GALNT13_ + 0.014817826 * Exp_IKBKGP1_ + 0.01969116 * Exp_SLC1A4_ + 0.019902233 * Exp_RP1_(2).

ROC analysis was used to test the predictive capacity of the gene-signature in the training and validation cohorts, and AUC values of 0.842 and 0.75 were obtained from the respective cohorts (Fig. 2C-D).

### Identification of hub genes associated with the obtained gene-signature and GRE

**Fig. 3:**
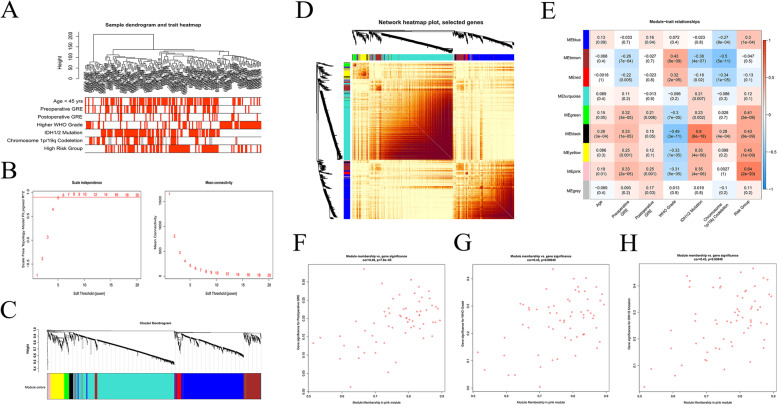
Identification of hub modules associated with gene-signature and postoperative seizure occurrence. **A**, Sample dendrogram and heatmap of the training cohort. **B**, Estimating soft-thresholding powers. The *β* power had R^2^ ≥ 0.90 (red line). **C** Cluster dendrograms, **D** heatmap, and **E** module-trait relationships plot representing the eight modules identified in the study. MEpink module had the strongest association with gene-signature and postoperative seizure occurrence. **F**–**H** Correlations between membership in the MEpink module and gene significance for preoperative GRE history, tumor grade, and IDH1/2 mutation

WGCNA was performed to identify hub genes associated with preoperative GRE history, postoperative seizure occurrence, and the obtained gene-signature. Previously reported risk factors for GRE, including age, WHO grade, IDH1/2 mutation status, and chromosome 1p/19q codeletion status were also included in the analysis (Fig. 3A). WGCNA was conducted using the top 5000 genes with the largest variance, and the soft-threshold power was set to 5 with scale free R^2^ ≥ 0.9 (Fig. 3B). A total of eight co-expression modules were identified (Fig. 3C). The topological overlap matrix constructed from the top 1000 genes revealed a strong co-expression of genes in these modules (Fig. 3D). Correlations between the eight modules and the included variables were analyzed (Fig. 3E). The MEpink module had the most significant positive correlation with higher gene-signature-derived risk score (*r* = 0.64, *p* = 2e-20), preoperative GRE (*r* = 0.33, *p* = 2e-05), and postoperative seizure occurrence (*r* = 0.25, *p* = 0.001) (Fig. 3E). This module was also correlated with IDH1/2 mutation (*r* = 0.35, *p* = 4e-06) and higher WHO grade (WHO grade 4, *r* = -0.31, *p* = 5e-05). Genes in the MEpink module (62 genes) showed similar correlations (Fig. 3F-H).

**Fig. 4:**
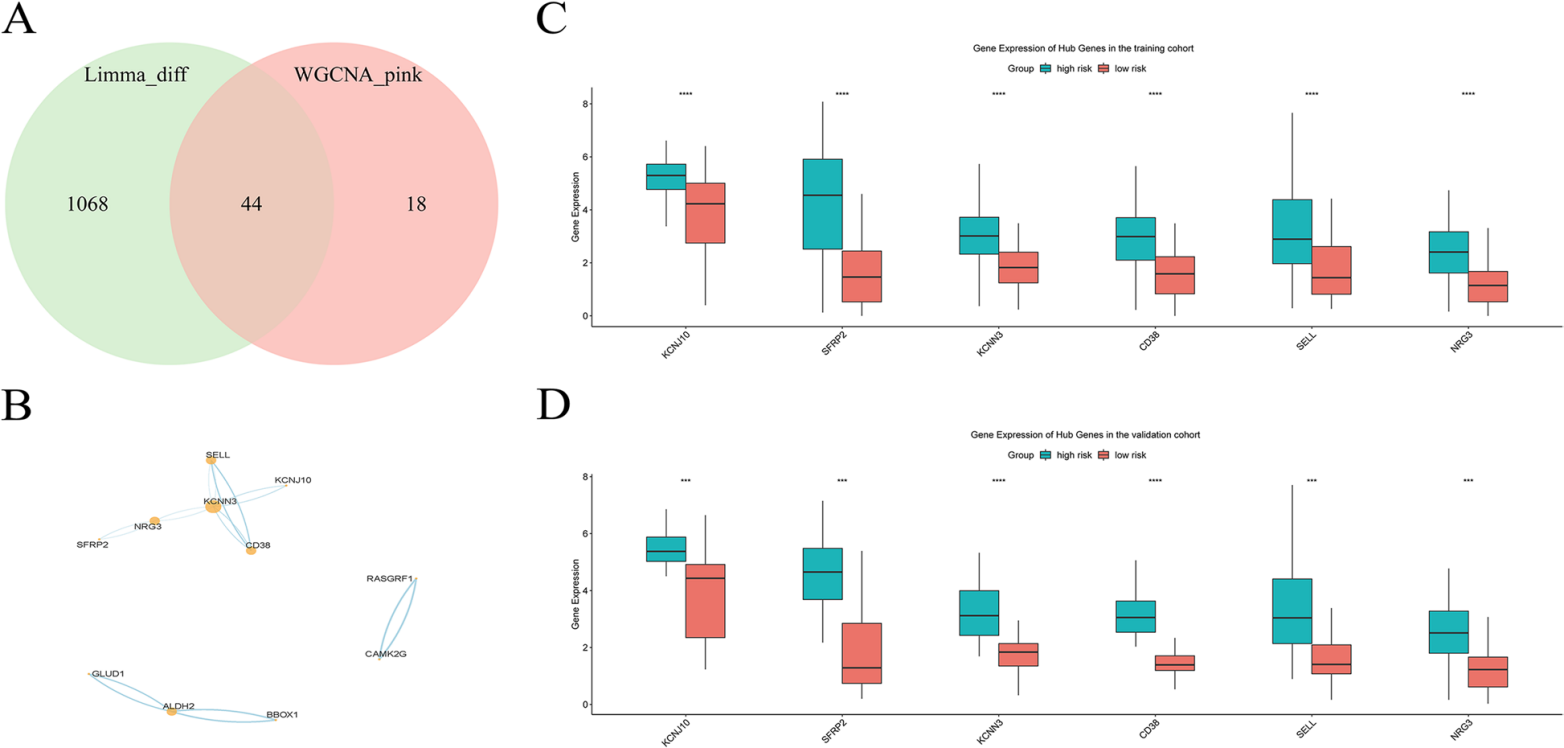
Identification and evaluation of hub genes associated with gene-signature. **A**, Venn diagram showing that 44 differentially expressed genes in the high-risk group overlapped with genes in the MEpink module. **B**, Three modules and six hub genes with close interactions were identified from the 44 overlapping genes through PPI network analysis. **C**-**D**, The expression of the six hub genes were significantly upregulated in the high-risk group in both the training and validation cohorts

Subsequent analysis of DEGs between patients with high and low gene-signature-derived risk scores was conducted to generate a new set of RDEGs. A total of 1112 RDEGs were upregulated in the high-risk group, with 44 of them overlapping with genes in the pink module (Fig. 4A). PPI network analysis identified a densely connected module consisted of six genes (Fig. 4B, Table 3). The expression of these genes was also upregulated in patients with gene-signature-derived high risk scores both in the training and validation cohorts (Fig. 4C-D).

**Table 3:** Main upregulated genes associated with GRE in high-risk group

Gene Name	Gene Title	Gene Function	Gene Expression		*p*-value
			High	Low	
CD38	CD38 molecule	Signal transduction	9.37	2.98	8.20858E-09
KCNJ10	Potassium inwardly rectifying channel subfamily J member 10	ATP-activated inward rectifier Potassium channel activity	41.13	21.04	4.92146E-10
KCNN3	Potassium calcium-activated channel subfamily N member 3	Small conductance calcium-activated potassium channel activity	10.04	3.12	4.31468E-09
NRG3	Neuregulin 3	Modulation of chemical synaptic transmission	5.55	1.64	3.05726E-10
SELL	Selectin L	Calcium ion binding	26.01	5.64	0.000682892
SFRP2	Secreted frizzled related protein 2	Receptor ligand activity	46.26	4.58	1.09581E-08

*GRE* glioma-related epilepsy

### Construction of an integrated prediction model for the occurrence of postoperative seizure

**Fig. 5:**
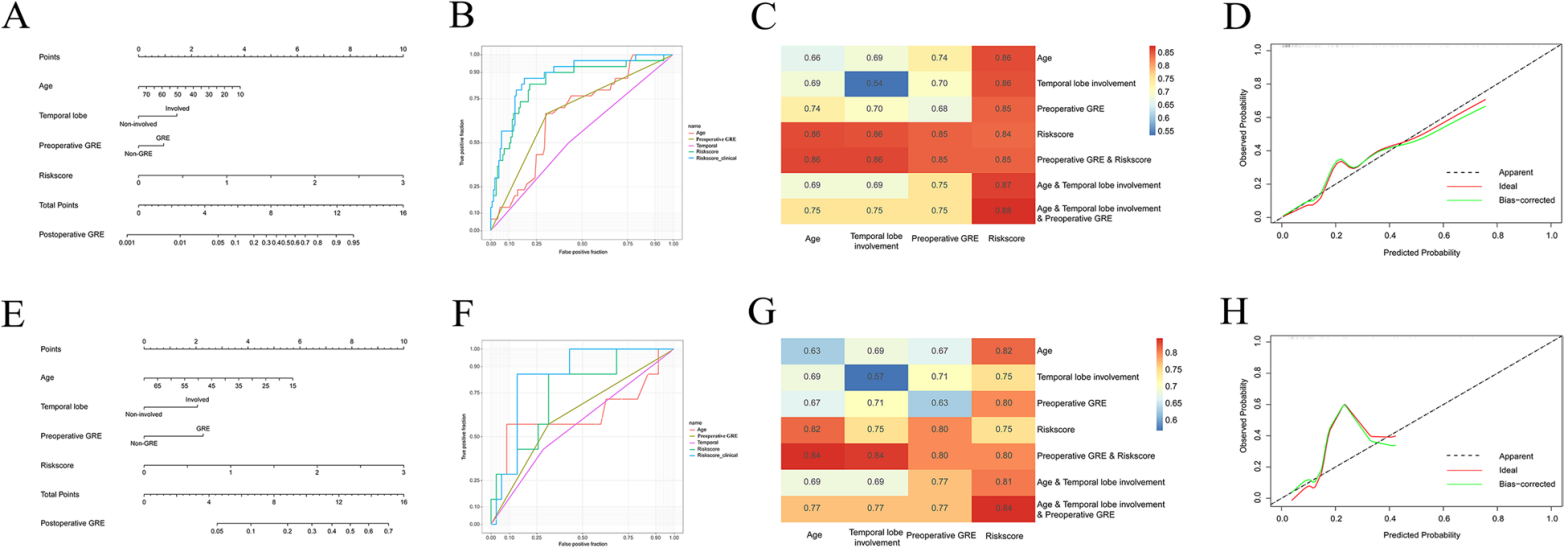
Construction and estimation of a predictive model for postoperative seizure occurrence in patients with diffuse high-grade gliomas (DHGGs). **A**, **E**, Nomograms showing that age, temporal lobe involvement, preoperative GRE, and gene-signature were included in the predictive model. **B**-**C**, **F**-**G**, Receiver operating characteristic (ROC) analysis showed that the predictive model had higher area under the curve (AUC) values compared with individual or combined variables. **D**, **H**, Calibration curves of a predictive model for postoperative seizure occurrence in patients with DHGGs

Multivariate logistic regression analysis was used to identify variables for constructing the postoperative seizure occurrence prediction model. Age (Odds Ratio [OR] = 0.944, *p* = 0.046), temporal lobe involvement (OR = 5.414, *p* = 0.011), preoperative GRE (OR = 4.120, *p* = 0.047), and higher risk scores (OR = 17.766, *p* < 0.001) were independently associated with postoperative GRE (Table 4). A predictive nomogram for postoperative seizure occurrence was generated using these four variables (Fig. 5A). The nomogram showed the weight of each variable and showed that the gene-signature-derived risk score was critical in the prediction of postoperative seizure occurrence. To further determine the role of gene-signature-derived risk scores in the model, ROC analysis was used to calculate the predictive performance of prediction models generated using different combinations of the four variables. The AUC was 0.75 when the model was generated using the three clinical variables, but increased to 0.88 when gene-signature-derived risk scores were added to the model (Fig. 5B-C). The calibration curve also showed good agreement between the predicted (red line) and observed (black dot line) probabilities (Fig. 5D). Finally, we evaluated the predictive capacity of our model using the validation cohort and obtained an AUC of 0.84 (Fig. 5E-H).

**Table 4:** Multivariable analysis of risk factors for postoperative seizure occurrence

Variable	*p*-value	OR	95% CI
Age	0.046	0.944	0.892–0.990
Temporal lobe involvement	0.011	5.414	1.480–19.804
Preoperative GRE	0.047	4.120	1.017–16.688
Risk-score	< 0.001	17.766	4.756–66.350

*OR* odds ratio, *CI* confidence interval, *GRE* glioma-related epilepsy

## Discussion

### Risk factors and prediction of postoperative seizure occurrence in patients with glioma

Postoperative GRE is a disturbing complication to both patients and treating physicians. Most neurosurgeons routinely prescribe postoperative AED prophylaxis for patients with glioma, even though their ability to reduce seizures is not clear [20, 21] and current research evidence and official guidelines do not support their administration [22, 23]. One potential root cause for this contradiction is the differences of seizure susceptibility among patients, for those with a high possibility of postoperative seizures, more aggressive AED prophylaxis maybe beneficial rather than current routine prophylaxis. Thus, studies on risk factors and predictors of postoperative seizure are important for personalized management of patients with glioma. Significant progress has been made in the study of patients with low-grade gliomas. Many clinical characteristics, including younger age, longer history of preoperative seizure, presenting as focal epilepsy, and sub-total resection, have been identified as risk factors for poor seizure control [24–26]. We previously developed an accurate RNA-seq-based model for predicting postoperative seizure outcomes in patients with diffuse low-grade gliomas [10].

This is the first study to generate a predictive gene-signature for postoperative seizure occurrence in patients with DHGG. We further developed an integrated prediction model using both gene expression and clinical data. Age, temporal lobe involvement, preoperative GRE history, and gene-signature-derived risk scores were identified as independent risk factors for postoperative seizure occurrence and included in the model. Of these, gene-signature-derived risk score was the most significant. Age, temporal lobe involvement, and preoperative GRE have also been confirmed as predictors of postoperative seizures by previous studies [9, 25–28]. Overall, the integrated prediction model showed good accuracy and stability. Given that patients who were excluded from this study differed from included patients in many aspects, the current integrated prediction model was statistically mainly applicable in DHGGs patients with relative longer survival. And the model can promote the prevention of seizures in these patients, improving their quality of life. However, limited by the conditions of enrolled patients, our model cannot provide sufficient information to avoid AEDs abuse in patients who may not need anti-epileptic treatment at all. Further prospective studies are needed to prompt rational use of AEDs.

### Potential mechanisms underlying GRE

GRE and glioma are comorbidities and share common pathogenic mechanisms. In this study, we explored the mechanisms underlying GRE and discovered that ion channel and immune system dysfunctions may play a major role in the epileptogenesis of GRE.

Epilepsy is correlated with changes in neuronal excitation/inhibition balance. Ion channel malfunctions can influence the extracellular concentrations of ions and neurotransmitters, inducing neuronal hyperexcitability that lead to seizures [29]. Altered ion channels are also associated with the proliferation and invasion of glioma cells [30]. In this study, functional enrichment analysis revealed that ion channel activities (especially potassium ion channels) were overactivated in patients with GRE. This may decrease interneuron activity and lead to disinhibition, resulting in seizures [31]. Moreover, pathway analysis showed that activities of the glutamatergic synapse and cAMP signaling pathways were enhanced in patients with GRE. Both pathways have been positively correlated with increased neuronal excitability [32, 33].

The correlation between immune dysfunction and glioma is widely accepted. Moreover, the relationship between immune dysfunction and epilepsy has been studied intensively, and the 2017 International League Against Epilepsy seizure classification identified immune group as a major etiological category for epilepsy [34, 35]. In our study, analysis of the immune cell infiltration landscape revealed an increase in the relative proportion of monocytes and a decrease in the relative proportion of M0 macrophages in patients with GRE. Both changes have been associated with better prognosis, representing lower tumor malignancy [36]. Thus, we inferred that GRE and tumor malignancy may be negatively correlated. Berendsen et al. found that epilepsy is an independent prognostic factor for longer survival in patients with glioblastoma. They proposed that the prognostic effect cannot be explained solely by the early diagnosis, but that the distinct biological features and alterations in gene expression may constitute the underlying mechanism [37]. The negative correlation can also be explained from the perspective of the epilepsy network. An integrated brain network is necessary for seizure generation and propagation, but tumors with higher malignancy can lead to the destruction of the brain network, blocking the propagation of seizures [38].

### Potential therapeutic targets for GRE

Seven genes were selected from the 623 DEGs and used to generate a predictive gene-signature for postoperative seizure occurrence. Of these, *SLC1A4* was essential in the gene-signature (*β* = 0.019691). *SLC1A4* is a member of the Solute Carrier Family 1, which has a high-affinity for glutamate and is involved in its transport in the central nervous system [39]. It encodes ASCT1, an effective transporter of glutamate under low pH, and is active in acidic tumor microenvironments [40]. We postulated that the mechanism underlying the correlation between *SLC1A4* overexpression and GRE involved upregulated *SLC1A4* resulting in increased concentration of glutamate in the peritumoral microenvironment, leading to overexcitement of the surrounding neurons and induction of seizures. *SLC1A4* may be a therapeutic target for GRE.

Additionally, WGCNA, RDEG identification, and PPI analysis were performed to identify additional potential therapeutic targets for GRE. Of the final six selected genes, the expression of *KCNJ10*, which encodes the inward-rectifier potassium channel Kir4.1, was correlated with the epileptogenesis of GRE. It has been reported that gain-of-function mutations in *KCNJ10* may lead to hyperexcitability through multiple unconfirmed mechanisms [31]. However, an animal model study suggested that reduced Kir4.1 activity could increase susceptibility to seizures by reducing potassium and glutamate buffering [41]. We inferred that upregulated *KCNJ10* expression in patients with GRE was more likely a compensatory measure for cell hyperexcitability rather than the cause of GRE [42].

## Limitations

This study is limited by its retrospective nature and relatively small sample size, and the prediction model needs to be further validated in large-scale prospective studies. The proposed mechanisms and therapeutic targets also need to be verified.

## Conclusions

We used RNA-seq data to propose a seven-gene signature for predicting the occurrence of postoperative seizures in patients with DHGGs. We then developed a prediction model including the identified gene-signature and clinical characteristics of the patients. Finally, we examined the potential mechanisms underlying GRE. The findings of this study may contribute to the development of personalized management strategies for patients with DHGGs and improve our understanding of the mechanisms underlying GRE in these patients. Further prospective validation of the model, ideally a randomized controlled trial of prophylactic AEDs vs no prophylaxis, in DHGGs patients with high-risk of postoperative GRE is still needed.

## Supplementary Information

**Additional file 1:** **Supplementary Fig 1.**Functional enrichment of differentially expressed genes (DEGs) associated withpreoperative GRE history. Mainly enriched molecularfunctions of upregulated (A) and downregulated (C) DEGs. Mainly enrichedpathways of upregulated (B) and downregulated (D) DEGs.

## Data Availability

The datasets used and/or analyzed during the current study are available from the corresponding author on reasonable request.

## Abbreviations

DHGGs: Diffuse high-grade gliomas
AEDs: Anti-epileptic drugs
DEGs: Differentially expressed genes
GRE: Glioma-related epilepsy
ROC: Receiver operating characteristic
LASSO: Least absolute shrinkage and selection operator
AUC: Area under the curve
GO: Gene Ontology
KEGG: Kyoto Encyclopedia of Genes and Genomes
GSEA: Gene Set Enrichment Analysis
WGCNA: Weighted correlation network analysis
RDEGs: DEGs based on risk scores
PPI: Protein-protein interaction
OR: Odds Ratio
