# Supplementary material for: Development of an integrated predictive model for postoperative glioma-related epilepsy using gene-signature and clinical data
Source: BMC Cancer. 2023 Jan 11;23:42. doi: 10.1186/s12885-022-10385-x (PMC9835377; doi:10.1186/s12885-022-10385-x)
Supplement: Supplementary file 1 — Additional file 1: Supplementary Fig 1.Functional enrichment of differentially expressed genes (DEGs) associated withpreoperative GRE history. Mainly enriched molecularfunctions of upregulated (A) and downregulated (C) DEGs. Mainly enrichedpathways of upregulated (B) and downregulated (D) DEGs. [file 12885_2022_10385_MOESM1_ESM.docx]

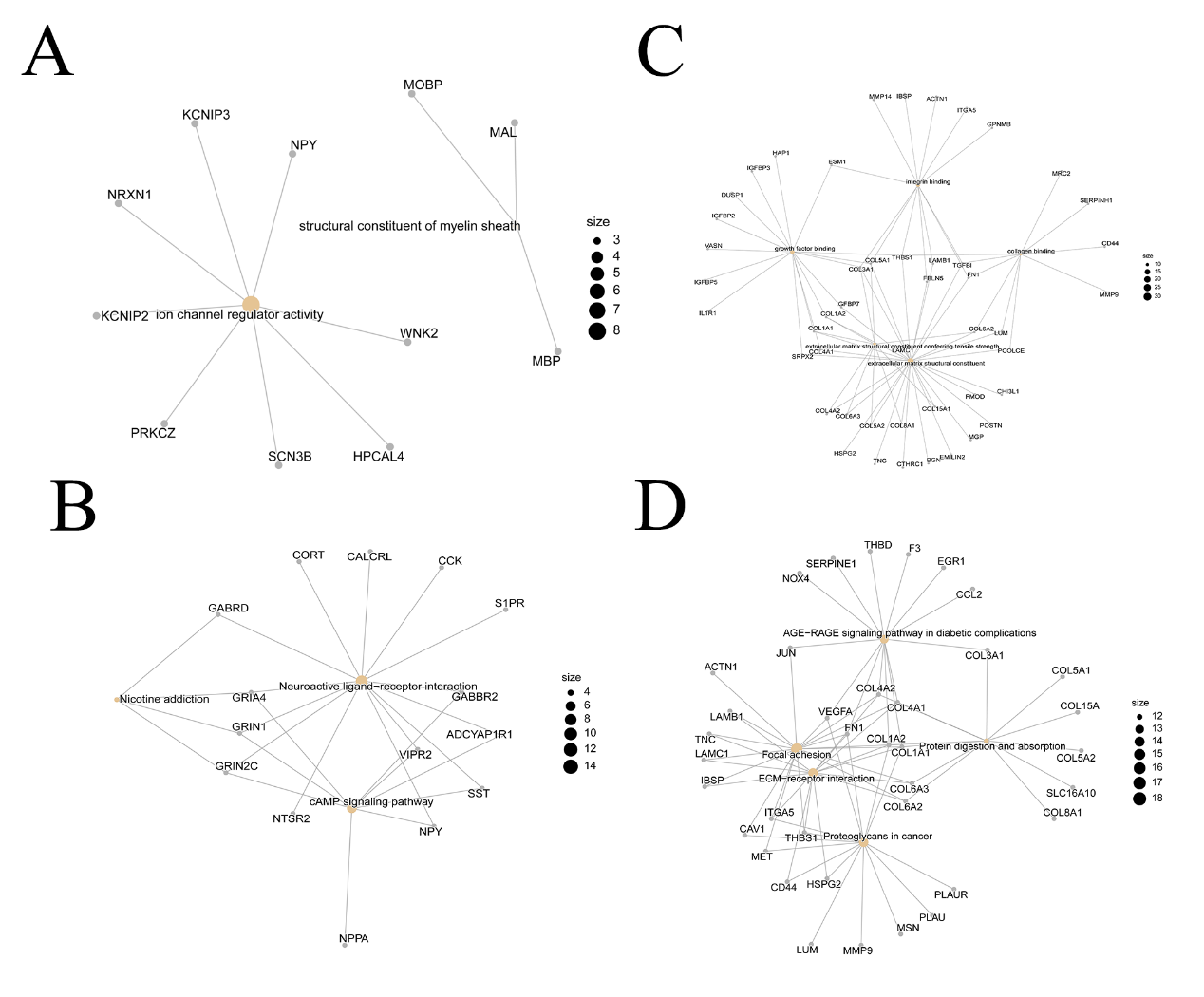


**Supplementary Fig 1. Functional enrichment of differentially expressed genes (DEGs) associated with preoperative GRE history.** Mainly enriched molecular functions of upregulated (A) and downregulated (C) DEGs. Mainly enriched pathways of upregulated (B) and downregulated (D) DEGs.
